# Differential Bilateral Primary Motor Cortex tDCS Fails to Modulate Choice Bias and Readiness in Perceptual Decision Making

**DOI:** 10.3389/fnins.2018.00410

**Published:** 2018-06-18

**Authors:** Esin Turkakin, Seda Akbıyık, Bihter Akyol, Ceren Gürdere, Yusuf Ö. Çakmak, Fuat Balcı

**Affiliations:** ^1^Timing and Decision Making Lab, Department of Psychology, Koç University, Istanbul, Turkey; ^2^Cakmak Lab, Department of Anatomy, School of Biomedical Sciences, University of Otago, Dunedin, New Zealand; ^3^Brain Health Research Centre, Dunedin, New Zealand; ^4^Medical Technologies Centre of Research Excellence, Auckland, New Zealand; ^5^Koç University Research Center for Translational Medicine, Koç University, Istanbul, Turkey

**Keywords:** transcranial direct current stimulation (tDCS), primary motor cortex (M1), perceptual decision making, drift diffusion model, computational modeling

## Abstract

One of the critical factors that guide choice behavior is the prior bias of the decision-maker with respect to different options, namely, the relative readiness by which the decision-maker opts for a specific choice. Although previous neuroimaging work has shown decision bias related activity in the orbitofrontal cortex, intraparietal sulcus (IPS) and dorsolateral prefrontal cortex, in a recent work by Javadi et al. (2015), primary motor cortex was also implicated. By applying transcranial direct current stimulation (tDCS), they have revealed a causal role of the primary motor cortex excitability in the induction of response time (RT) differences and decision bias in the form of choice probability. The current study aimed to replicate these recent findings with an experimental design that contained a sham group to increase experimental control and an additional testing phase to investigate the possible after-effects of tDCS. The conventional decision outputs such as choice proportion and RT were analyzed along with the theory-driven estimates of choice bias and non-decision related components of RTs (e.g., motor implementation speed of choices made). None of the statistical comparisons favored the alternative hypotheses over the null hypotheses. Consequently, previous findings regarding the effect of primary motor cortex excitability on choice bias and response times could not be replicated with a more controlled experimental design that is recommended for tDCS studies (Horvath et al., 2015). This empirical discrepancy between the two studies adds to the evidence demonstrating inconsistent effects of tDCS in establishing causal relationships between cortical excitability and motor behavior.

## Introduction

Many of our routine decisions include relatively speedy judgments made based on a continuous stream of sensory information. These range from deciding if we have previously met a given person to deciding if an approaching cab is occupied or not. Such decisions are typically conceptualized as originating from the mental integration of sensory input depending on which of the two (or more) decision states it favors. In laboratory settings, these decisions are generally investigated via two-alternative forced choice (2AFC) tasks. In a typical 2AFC scenario, participants observe a sensory stimulus, decide which of the two predetermined states it best represents, and indicate their decision by pressing the corresponding response key. Critically, the data gathered from 2AFC tasks enable researchers to trace the observed decisions at the level of latent variables that bear psycho-mechanistic meaning. In the current study, we used transcranial direct current stimulation (tDCS) as a tool to investigate the potential causal roles of primary motor cortex activity in the modulation of such latent variables in a perceptual decision making task.

Capitalizing on the analytical tractability of behavioral data gathered from 2AFC tasks, a number of influential generative models can account for the decision accuracy along with the shapes of the associated response time distributions (Ratcliff, 1978; Usher and McClelland, 2001; Brown and Heathcote, 2008). These models can be thought as generative extensions of the Signal Detection Theory (SDT; Green and Swets, 1966), which constitutes a non-dynamic mechanistic approach. While SDT only accounts for changes in accuracy for the two choices, the generative models use RT distributions for both correct and error responses to estimate latent variables.

A prominent example of these generative models of choice behavior is the diffusion decision model (also known as the drift-diffusion model – DDM; for a review, see Ratcliff and Mckoon, 2008). The DDM assumes that sensory evidence is integrated at some rate continuously over time (drift rate) in a noisy fashion (diffusion) toward one of the two imaginary bounds (decision thresholds). These bounds represent the two alternative states that the sensory information could favor (i.e., hypotheses). Within the framework of this decision-theoretic approach, the rate of evidence integration reflects the strength of sensory information and the decision bounds correspond to the amount of evidence that needs to be accumulated before committing to a choice. These two core parameters of DDM estimated from the shape of the correct and error RT distributions, as well as their relative density, were shown to be sensitive to the signal-to-noise ratio in sensory information (e.g., higher drift rates for high signal-to-noise ratio; Balcı et al., 2011) and differential emphasis on speed vs. accuracy (e.g., higher thresholds when accuracy is emphasized; Bogacz et al., 2010). A number of neuroimaging and brain stimulation studies have implicated the fronto-parietal pathways for the modulation of the drift rate and the pre-supplementary motor area for the modulation of the decision thresholds (Mulder et al., 2014; Tosun et al., 2017; Berkay et al., 2018).

The other two core parameters of DDM are the starting point bias and non-decision related delays. The starting point (z, the location of the decision variable prior to evidence integration) represents the initial belief state regarding the two choices or the response bias of the decision-maker. For instance, one would expect higher prior probability, higher payoff, or lower cost associated with a decision to shift the starting point closer to the corresponding decision threshold (Simen et al., 2006; Rorie et al., 2010; Summerfield and Koechlin, 2010; Mulder et al., 2012; Hagura et al., 2017). Manipulations that are designed to induce a change in the bias parameter activate certain cortical and subcortical structures that are implicated in action-selection, motor planning and cognitive control such as OFC, DLPFC, IPS, and striatum (Forstmann et al., 2010; Mulder et al., 2014).

The non-decision related delay, another parameter of the decision-process is assumed to have two independent components: signal-detection time and delay associated with the motor manifestation of the decision made upon the threshold-crossing. This parameter was shown to be associated with the predictability of and thus the resultant preparation for the onset of stimulus presentation (Grosjean et al., 2001; Jepma et al., 2012). Compared to the drift rate and decision threshold modulation, the investigation of the neural mechanisms that underlie the modulation of these two parameters is relatively limited (Mulder et al., 2012).

The tDCS is a non-invasive brain stimulation technique, which modulates cortical excitability by delivering weak electrical currents (Nitsche and Paulus, 2000). This relatively accessible tool provides an opportunity to study the causal role of brain regions in decision processes. Through the electrodes that are positioned directly on the scalp, tDCS can induce a polarity-dependent effect on the membrane potential by manipulating the ion concentration inside and outside the membrane; while anodal stimulation increases neuronal excitability through depolarization, cathodal stimulation leads to hyperpolarization and thus results in a reverse effect (Nitsche and Paulus, 2000, 2011). Although tDCS has been utilized in a wide range of domains that include but are not limited to working memory (e.g., Fregni et al., 2005), conceptual processing (e.g., Lupyan et al., 2012), cognitive control (e.g., Wolkenstein and Plewnia, 2013), speech production (Marangolo et al., 2013), and visuomotor learning (e.g., Nitsche et al., 2003; Antal et al., 2004), conflicting findings in literature cast doubt on the validity and efficiency of this method in affecting behavior at least in healthy participants (for a review, see Horvath et al., 2015).

Motor behavior has been a common target for modulation using tDCS mainly for two reasons. First, the minimal variance across individuals in the orientation of neurons in the motor cortex and its proximity to the skull would increase the efficacy of electrical stimulation (Nitsche and Paulus, 2001). Second, the effect of tDCS on motor processes can be investigated through simple methods such as measuring the changes in the magnitude of TMS-induced motor evoked potentials (MEP) (e.g., Bastani and Jaberzadeh, 2013) or the performance in simple motor tasks (e.g., Boggio et al., 2006; Tanaka et al., 2009; Hummel et al., 2010). For instance, in a serial reaction time task, Nitsche et al. (2003) showed that anodal stimulation of primary motor cortex leads to faster responses committed by the hand contralateral to the stimulation site. As another example, Tanaka et al. (2009) measured the maximal pinch forces of the toe and anodal tDCS led to a transient increase in the pinch force.

In this study, we investigated whether the functional outcome of primary motor cortex modulation would generalize to other and more complex cognitive domains such as decision making. Specifically, we tested how modulating the excitability of the primary motor cortex in one vs. the other hemisphere via their anodal vs. cathodal stimulation affects the decision making at the level of both overt choice behavior (i.e., hand-choice and RT) and the latent variables that guide these behaviors (i.e., starting point bias, non-decision related delay, and drift rate).

This question was recently addressed at the level of behavioral decision outputs by Javadi et al. (2015) in a 2AFC paradigm. In each trial, participants were presented with rectangles that differed in their height-to-width ratios. The target rectangle was masked after 100 ms following stimulus onset. Following the mask (400 ms), a question mark appeared on the screen. The task of the participant was to report the orientation of the rectangle (right/left) by using their index fingers (right/left, respectively) as soon as they saw the question mark. Since the frequency of different height-to-width ratios was the same, the frequency of choices that are committed by either hand would be the same for a participant without bias. Javadi et al. (2015) aimed to disrupt this equivalence by modulating hand choice through tDCS. They documented that the montage of bilateral stimulation (excitatory on one side and inhibitory on the other) of the primary motor cortex resulted in a change in the frequency of choices committed by the hand contralateral to the stimulation site, inducing a bias in hand-choice. That is, the right-anodal/left-cathodal (rAlC) montage resulted in a trend of increased frequency of choices committed by the left hand, but there was no significant effect of the left-anodal/right-cathodal (lArC) montage. They also observed a modulatory effect of brain stimulation on the response times of the left hand (non-dominant hand in their sample) in correct trials. However, they did not investigate the effect of their brain stimulation manipulations on the latent decision-making processes, which provide a more decision theoretically grounded characterization of the presumed effects based on the unified analysis of the decision accuracy and associated response times (e.g., Tosun et al., 2017; Berkay et al., 2018).

Javadi et al. (2015) offered two accounts for how their experimental manipulation may have affected the decision parameters within the context of accumulation to bound models. One account is that the stimulation modulates the activity of not just M1 but a wider area encompassing the premotor cortex and parietal areas (Bikson and Rahman, 2013), and thus affects non-motor aspects of the decision such as perceptual evidence accumulation. The other account posits that the stimulation, by changing the excitability of the primary motor cortex, shifts the starting point of the decision particle toward the threshold associated with the hand contralateral to the stimulated site and away from the other hand. They speculated that their results are more in line with the latter account, but this remains untested through actual modeling of the behavioral data. Considering the above-mentioned effects of tDCS on motor tasks, we suggest that another potential target parameter not discussed by Javadi et al. (2015) could be non-decision time, since it reflects changes in the motor manifestation of decisions.

Based on the previous work by Javadi et al. (2015) outlined above, we had two conceptually interrelated sets of hypotheses at different levels of analyses. At the behavioral level, we predicted the anodal stimulation of the primary motor cortex (and cathodal stimulation of the corresponding area in the other hemisphere) to lead to faster RTs and bias toward the decision (in terms of choice proportion) associated with the contralateral hand. At the level of latent decision processes, we had three non-mutually exclusive hypotheses: we expected the stimulation to affect (1) the non-decision time (*t_0_*), meaning an effect on the motor processes that were not related to the decision, (2) and/or the starting point, meaning an effect on the choice bias (using SDT’s criterion setting as a proxy measure of this DDM variable due to our experimental design and poor model fit quality), (3) and/or the drift rate, meaning an effect on the rate of evidence accumulation process. For non-decision time, we expected a decrease for the stimulated hand and an increase for the inhibited hand. As to the starting point, we expected a shift toward the decision threshold associated with the stimulated hand and away from that of the inhibited hand. Finally, for drift rate, we expected an increase for the option associated with the stimulated hand and a decrease for the option associated with the inhibited hand.

## Materials and Methods

### Participants

An *a priori* power analysis (GLIMMPSE; Kreidler et al., 2013) indicated that three subjects in each of the eight cells (2 (montage) × 2 (hand) × 2 (stimulation), 24 participants in total) was needed to reach a 80% power of detecting at least half the mean difference with double the variability in RT that is reported in Javadi et al. (2015) (as estimated from Figure 4A in that paper). We chose to use a more conservative effect size in the power analysis in order to ensure that a potential failure to replicate findings of Javadi et al. (2015) did not result from the possible decrease in effect size and thus statistical power in our study due to peripheral factors such as the particular stimuli and the response deadline we imposed.

Twenty-four right-handed healthy volunteers with normal or corrected vision participated in the study (three in each group) (M_age_ = 20.3 ± 2.33, 14 females). We recruited the participants through an online announcement in a daily newsletter available to Koç University community. Individuals who had one of the following conditions were not included as participants: current or past neurological and psychological disorder in the individuals and their immediate families, metal implants, current psychoactive or allergy medication, or drug use in the past year. A further exclusion criterion was the presence of a perceptual or hand-choice bias in the first part of the task (see section “Procedure” for details). Participants were compensated with monetary reward for their participation in the study (up to a total of 40 TL for full participation in two sessions). All participants gave written informed consent in accordance with the Declaration of Helsinki, and the protocol was approved by the Committee on Human Research of the Koç University Institutional Review Board.

### Apparatus

The experiment was run on an iMac (60 Hz refresh-rate, 21.5 inch, 1920 pixels × 1080 pixels, AMD Radeon HD 4670, 256 MB graphics) and stimulus presentation and data collection were conducted in MATLAB 2015b with Psychtoolbox 3. Participants responded via a wired keyboard (Dell KB212 - B). We used NeuroConn DC-Stimulator PLUS with 5 cm × 5 cm electrodes encased in saline soaked sponge sleeves. We placed the electrodes on the motor cortex, centered on C3 and C4 sites according to the international 10-20 system (EEG cap model: g.Gamma, GTEC), with the cable attached along the superior-inferior axis.

### Procedure

**FIGURE 1:**
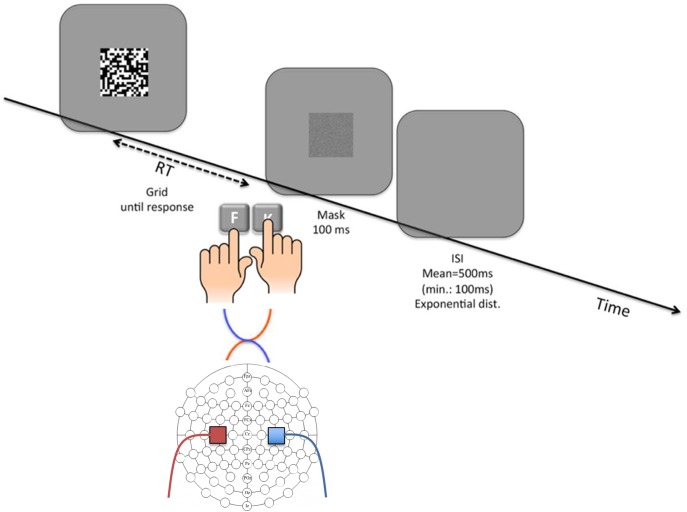
Illustration of an example trial of the grid-task and tDCS electrode montage. In each trial, the grid is presented until the participant initiates a response which is followed by the presentation of a random-noise mask. After a variable interval that is drawn from a left-truncated exponential distribution (*M* = 500 ms, lower limit = 100 ms), the next trial starts. The diagram depicts a left Anode right Cathode (lArC) montage where the anode on C3 provides the left primary motor cortex (and thus the right hand) with excitatory stimulation, and the cathode placed on C4 provides the right primary motor cortex (and thus the left hand) with inhibitory stimulation.

We used a perceptual decision making task, in which a 20 × 20 grid (12.5 cm × 12.5 cm, with participants at approximately 60 cm viewing distance) comprised an unequal number of black and white squares (52% of the squares in randomly assigned dominant color) was presented and participants were asked to decide whether black or white squares were more frequent on the grid. The responses were emitted by pressing the corresponding key using both hands placed on the keyboard; *F* with left hand and *K* with right hand. The key-color assignment (*F* corresponding to black-*K* corresponding to white and vice versa) were counterbalanced across participants and remained constant throughout both sessions for each participant. Participants were asked to respond within a deadline of 800 ms, responses after this deadline were labeled as late and did not count as correct responses in the feedback provided to the participants. Following the response, a random noise mask the same size as the grid was presented for 100 ms (see **Figure 1**). Since the stimuli disappeared only after participants executed a response, we masked the stimulus after the response to eliminate the visual trace of the previous stimulus particularly given its static nature. Following every 10th trial, participants received feedback indicating the total number of completed trials and the number of correct responses.

Participants attended two sessions which were separated by at least two days. Each session consisted of three 10-min-long grid-task phases, starting with a brief training of 40 maximum trials. Participants who passed the criterion of five timed responses given within the 800 ms limit skipped to the first phase of the experiment without completing all of the training trials. At the end of the first phase, a two-tailed binomial test with an alpha level of *p* = 0.01 was applied to determine a bias for a color (white or black) or a hand (right or left). Individuals with any type of bias (40% of the recruited participants) were not administered the rest of the experimental phases (see section “Discussion” for a detailed explanation of this exclusion criterion). In preparation for the tDCS, electrode placements were determined by 10–20 system EEG-caps. The electrode locations on the scalp and the sponge envelopes for the electrodes were dampened with 0.60% (w/v) NaCl solution in order to provide the necessary conductance. Electrodes were attached by the rubber bands to the C3 location for left and C4 for right motor cortex (see **Figure 1** for the montage illustration).

**FIGURE 2:**
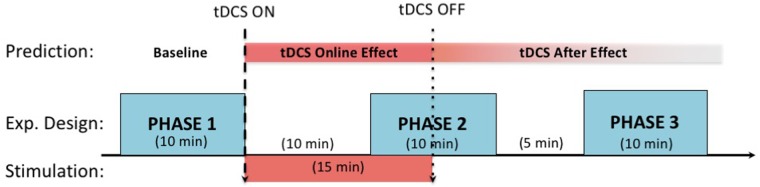
Session procedure along with the expected timelines for the stimulation effects. In each phase, participants complete a 10 min long grid-task. tDCS stimulation starts at the end of the first phase and lasts for 15 min. Following the 10th minute of the stimulation, the second phase starts. After a 5-min break, the third phase starts, after which the session ends. The first phase serves as the baseline for assessing the online (second phase) and offline after effects (third phase) of tDCS.

Participants were randomly assigned to active or sham stimulation conditions in a randomized, double-blind fashion. For the active stimulation group, 1.5-mA direct current was administered for 15 min with 10-s fade-in and fade-out durations. The sham stimulation group received the same stimulation intensity for 30 s with the same fade-in and fade-out durations, but saw an identical display on the stimulator indicating a 15-min stimulation. The experimenters were blind to the stimulation condition as this was determined by a pre-assigned random number that corresponded to either an active or sham stimulation with identical displays on the stimulator. The second phase started in the last 5 min of stimulation, and the final phase was carried out following the second phase, with a 5 min break in between (see **Figure 2** for the experimental procedures within a session). The same procedure was followed in the second session except for the switching of electrode placement (right anodal and left cathodal to left anodal and right cathodal, or vice versa), the order of which was counterbalanced across participants.

### Data Analysis

#### Behavioral Outcomes

The two behavioral outcomes of interest were change in hand choice and response time between test phases. We quantified hand choice as the percentage of choices committed with the left hand in all trials. The differences in hand choice between pairs of phases were then used as the dependent variable in order to make our analyses comparable to those of Javadi et al. (2015). Similarly, the differences in median response times between pairs of phases were used to investigate the effect of tDCS stimulation on response speed. In addition to the conventional frequentist analysis of variance (ANOVA) tests, we used Bayesian ANOVA (Rouder et al., 2012) to test all our behavioral hypotheses, which allowed us to quantify the extent to which the null hypothesis is favored over the alternative hypotheses, given our data. We provide both *p* values and Bayes Factors for each analysis for ease of comparison. For the Bayesian analyses, we use Bayesian Model Averaging and we report the inverted BF_inclusion_ values (1/ BF_inclusion_, reported as BF_exclusion_) for the effects of interest, indicating the factor by which the *exclusion* of the effect from the model is supported by our data (see Wagenmakers et al., 2017 for further detail). We used the JASP 0.8.1.2 for all tests (JASP Team, 2018).

In order to compare our results directly to those of Javadi et al. (2015), we first conducted the same analyses on our data from the first two phases of the participants that received active stimulation, as this constituted the extent of their experimental setup. For the comparison of hand choice effects, we conducted a one-way repeated-measures ANOVA with electrode montage (lArC and rAlC) as the within-subjects factor. The dependent variable was the change in left hand choice percentage from the first test phase (baseline) to the second (active stimulation). In order to match their response time analysis, we conducted a 2 × 2 repeated-measures ANOVA with electrode montage (lArC and rAlC) and hand (right and left) as within-subject factors and change in median response times from the first phase to the second as the dependent variable. We also conducted the same analysis with correct response trials only, following Javadi et al. (2015) as they report no significant effect with all trials but find an effect with correct trials.

Besides replicating Javadi et al. (2015)’s analyses, we ran additional analyses to compare the active/sham stimulation groups and to investigate the offline effect of tDCS on hand choice and response times. For each pair of phases (Phases 1-2, 2-3, and 1-3), we conducted a 2 × 2 mixed factor ANOVA with electrode montage (lArC and rAlC) as within-subjects and stimulation condition (Active or Sham) as between-subjects factors for hand choice effects, and a 2 × 2 × 2 mixed factor ANOVA with electrode montage (lArC and rAlC) and hand (right and left) as within subjects and stimulation condition (Active or Sham) as between-subjects factors for response time effects.

For all analyses, we only report the significance tests of the highest level interactions, as only these pertain to the potential modulatory effect of stimulation on behavior. When testing the effect of stimulation on response times, for example, we would expect different stimulation setups to change the behavior of the two hands differentially, and only for the group that actually received the stimulation. This calls for testing the three-way interaction of condition, montage, and hand, as any lower level interactions or main effects were not targeted through stimulation.

#### Decision Model Parameters

In order to see how our data fit a model where tDCS affected the motor processes unrelated to the decision (*t_0_*; non-decision time) and the perceptual evidence accumulation efficiency (*v*; drift rate) separately for responses committed with either hand, we split the data of each individual into two by response hand (key press) and used accuracy coding where the upper threshold corresponded to correct responses and the lower threshold corresponded to errors. Using hierarchical Bayesian estimation of DDM (Wiecki et al., 2013), we tested three nested models. In the more theoretically constrained models (Model I and II), only *t_0_* and only *v*, respectively, varied with stimulation condition (as the between-subject factor; 2 levels) and three experimental phases in both electrode montages (as the within-subject factor; 6 levels). In the exploratory model (Model III) testing whether drift rate was also affected alongside non-decision time, both *t_0_* and *v* (drift rate) varied with the same factors. We compared these models to the null model (Model 0) where all parameters varied for each participant but not with any other factor.

Deviance Information Criterion (DIC) was used for model selection. A ΔDIC of at least -10 was considered as sufficient improvement over the reference model. All models were fit using 5000 samples, with the first 20 draws discarded as burn-in. We also discarded trials with response times faster than 100 ms as premature responses. The models assumed that a uniformly distributed 2.5% of the remaining trials were generated by processes unrelated to the decision (e.g., distraction) and thus excluded them from the fitting as outliers. We evaluated goodness of fit via posterior predictive checks (comparing 95% credible intervals of data generated from model outcomes and the observed data) and visual inspection of convergence metrics (sample distributions, traces, and auto-correlations). For hypothesis testing, we compared the posterior distributions of the parameters under different conditions. The comparisons yielded the probability (*P*) of a parameter value in the posterior being higher than the corresponding value in the other posterior for each sample, and we considered any probability higher than 0.95 to be a sufficient difference between the posterior distributions as per convention.

We also investigated models where tDCS affected starting point bias (*z*) toward the hand that corresponded to the stimulated motor cortex. The models we used to estimate non-decision time used accuracy-coded data split by response hand, as HDDM cannot estimate two separate *t_0_*s from the full data. This setup was not suitable for starting point bias models, as bias toward one of the boundaries does not make theoretical sense with accuracy-coded data where the boundaries stand for the correct and incorrect responses. The three main approaches we took with HDDM were (1) splitting the accuracy coded data into two by stimulus type (according to which hand was mapped to the correct response), where a bias toward the upper boundary for one stimulus (i.e., right hand for right-hand stimuli) would be observed as a bias toward the lower boundary in the other stimulus (i.e., right hand for left-hand stimuli), (2) modeling the full data without splitting and using a link function (converting z to 1-*z* for one of the stimulus types) to force the complementarity that was assumed in Approach 1, and (3) using the “Stimcoding” module of HDDM which automatically reverses the *z* to 1-*z* for one of the stimuli and thus implements Approach 2 internally. Approach 3 is actually limited to between-subjects models and thus unfit for our design, but we wanted to try it to check whether it matched the outcomes of Approach 1 and 2. The main assumption for Approach 1 was that the *z* estimates for the two types of stimuli would be complementary (sum to approximately 1), as was explained above. Our model did not meet this criterion, as the starting point was estimated to be roughly the same (∼0.43) for both right-hand and left-hand stimuli, indicating a bias not toward a particular hand but toward the incorrect response in both cases. Approach 2 failed as the MCMC chains did not converge, rendering the estimates unreliable and indicating that forcing this property onto the data would not be suitable. Approach 3 did not find a significant bias toward either hand, and thus did not match the outcome of the other two approaches. Since testing our predictions through these models would not be valid or informative, we do not report any comparisons from these models. Instead, we use Signal Detection Theory to quantify and compare decision bias under different conditions.

#### Signal Detection Theory Outcomes

In order to see if stimulating and inhibiting the primary motor cortices resulted in changes of sensitivity or preference for either hand, we resorted to metrics from Signal Detection Theory adapted to 2AFC paradigms. In this analysis, stimulus mapped onto the right hand response was treated as signal and stimulus mapped onto the left hand response was treated as noise. Consequently, correct right-hand responses were Hits, incorrect right-hand responses were Misses, correct left-hand responses were Correct Rejections, and incorrect left-hand responses were False Alarms. D-prime was calculated as $d^{\prime }=\frac{z(Hit)-z(False\;Alarm)}{\sqrt{2}}$ and criterion was calculated as $\beta =-\frac{z(Hit)+z(False\;Alarm)}{2}$ (Macmillan and Creelman, 2004). The effects of stimulation condition, electrode montage, and experimental phase on criterion setting and d-prime were then tested using a 2 × 2 × 3 mixed factor ANOVA design.

## Results

### Behavioral Outcomes

#### General Descriptives

**FIGURE 3:**
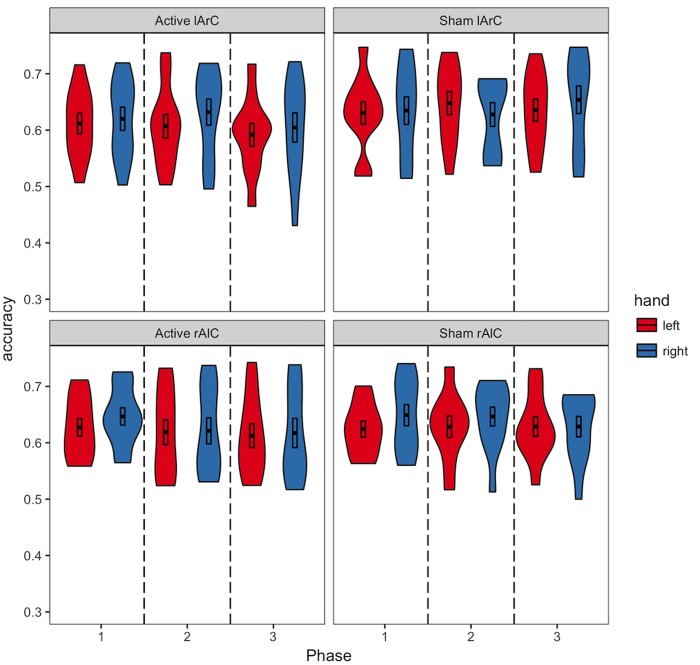
Distribution of the accuracies of participants in different conditions. The black bars in the middle represent 1 standard error above and below the mean and the thick line dividing the bars represents the mean. We did not have any explicit expectations regarding changes in accuracy due to tDCS.

**FIGURE 4:**
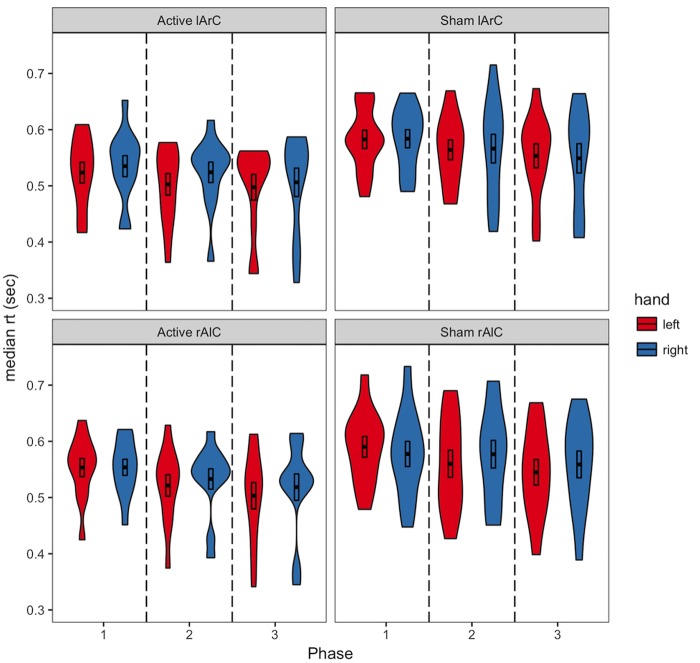
Distribution of median RTs of active and sham participants for responses with left/right hand as a function of different phases and montage conditions. The black bars in the middle represent 1 standard error above and below the mean and the thick line dividing the bars represents the mean. Regarding the online effects, anodal stimulation of either hemisphere was expected to result in a difference between the first and second phase for the responses with the contralateral hand (e.g., a decrease in median RT of left hand response for rAlC stimulation condition), only for the active stimulation group. Regarding the offline effects, a similar pattern was expected as a change between the first and third phase. The reverse was expected for the cathodal stimulation.

**FIGURE 5:**
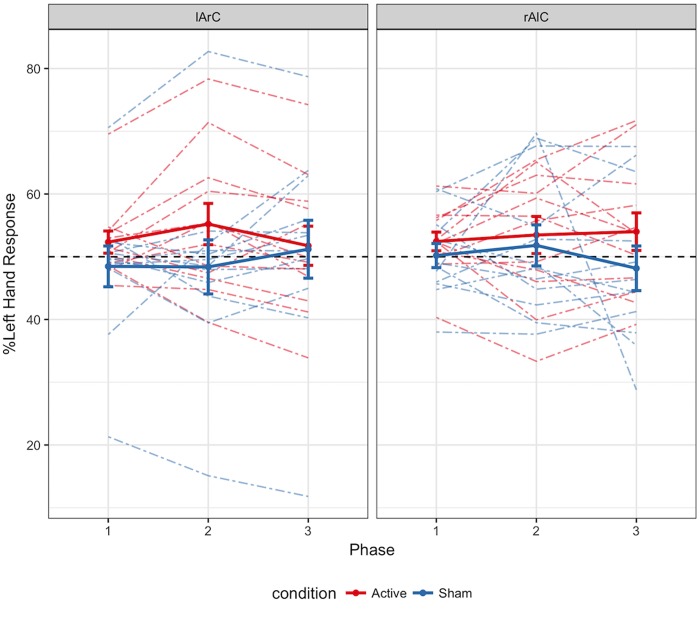
Distribution of percentage of left hand responses as a function of phase, montage, and stimulation conditions. The data of each participant in different phases are connected via colored dashed lines. The solid lines represent the average for active and sham participant groups. Error bars represent ± 1 SE. The black dashed line denotes the point where the two hands were used equally. Higher values on the y axis indicate an increase in the responses initiated with the left hand. Between first and second phases, an increase in left hand response was expected for the active anodal stimulation of the right hemisphere (rAlC) and a decrease in left hand response was expected for the active cathodal stimulation of the right hemisphere (lArC).

Participants averaged 63.2% accuracy with a response time of 554 ms across all conditions (see **Figures 3, 4** for how accuracy and median RTs varied with the conditions). On average, the participants completed over 2200 trials each, missed the 800 ms response deadline in 3.9% of the trials, and responded to 48.6% of the trials with their left hand across all conditions (see **Figure 5** for how left-hand choice percentage varied with the conditions).

#### Replication of the Analyses in Javadi et al. (2015)

To assess whether we could successfully replicate the first experiment of Javadi et al. (2015), we conducted the statistical analyses reported in that study on a subset of our data that corresponded to their dataset (only the first and second test phases of participants who received active stimulation). We did not find an effect of montage condition on hand choice [*F*(1,11) = 0.29, *p* = 0.60, *η^*2*^* = 0.03; *BF_exclusion_* = 2.33], and on response times [*F*(1,11) = 0.55, *p* = 0.48, *η^*2*^* = 0.05; *BF_exclusion_* = 2.81]. We did not detect an interaction between electrode montage and response hand with respect to their effects on RT either [*F*(1,11) = 0.01, *p* = 0.93, *η^*2*^* = 0.001; *BF_exclusion_* = 6.29]. We also repeated the response time analysis for correct responses only, as Javadi et al. (2015) report that they could only detect the RT effect in that subset of trials. However, we found neither a main effect of electrode montage [*F*(1,11) = 0.51, *p* = 0.49, *η^*2*^* = 0.04; *BF_exclusion_* = 3.07] nor an interaction of montage and hand [*F*(1,11) = 0.002, *p* = 0.96, *η^*2*^* < 0.001; *BF_exclusion_* = 7.30] on correct response times. Consequently, our study fails to replicate the results of the corresponding comparisons in Javadi et al. (2015).

#### Online Effects of tDCS (Phase 2–Phase 1)

The effect of interest, a three-way interaction of condition, montage, and hand, was not found to be significant [*F*(1,22) = 0.53, *p* = 0.48, *η^*2*^* = 0.02; *BF_exclusion_* = 250] with respect to the RT differences from the first phase to the second. Similarly, for changes in hand choice, the two-way interaction of electrode montage and condition was non-significant [*F*(1,22) = 0.79, *p* = 0.39, *η^*2*^* = 0.03; *BF_exclusion_* = 7.69].

#### Offline Effects of tDCS (Phase 3–Phase 1 and Phase 3–Phase 2)

The change in RT from the first and second phases to the third phase did not depend on the three-way interaction of montage, hand, and condition [for difference from baseline: *F*(1,22) = 0.02, *p* = 0.90, η^*2*^ = 0.001; *BF*_exclusion_ = 333; for difference from the online phase: *F*(1,22) = 1.00, *p* = 0.33, η^*2*^ = 0.04; *BF*_exclusion_ = 500]. Similarly, the two-way interaction of condition and montage effects were non-significant for changes in hand choice from both the first [*F*(1,22) = 1.97, *p* = 0.18, η^*2*^ = 0.08; *BF*_exclusion_ = 4.02] and the second [*F*(1,22) = 4.00, *p* = 0.06, η^*2*^= 0.15; *BF*_exclusion_ = 1.25] phases to the final phase. *Post hoc* tests reveal a significant difference between the active (*M* = -3.46, *SD* = 4.30) and sham (*M* = 2.82, *SD* = 5.86) groups for change in left hand use from the second to the third phase under lArC stimulation [*t*(22) = -2.99, *p* = 0.01; *BF*_10_ = 6.92], but not under rAlC stimulation (*M*_Active_ = 0.53, *SD*_Active_ = 6.53, *M*_Sham_ = -3.65, *SD*_Sham_ = 13.09; *t*(22) = 0.99, *p* = 0.33; *BF*_01_ = 1.88). The change in the active stimulation group is in the expected direction, but the effect in the sham group, which contributes to the significant difference, is not expected at all since this group did not actually receive any stimulation.

#### Model Outcomes

**FIGURE 6:**
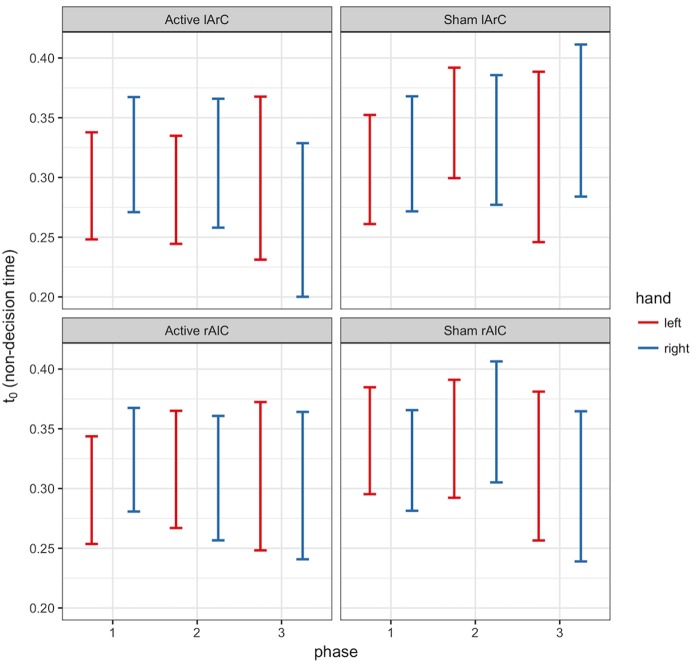
95% credible intervals of non-decision time (*t*_0_) posterior distributions of left (red) and right (blue) hands for different phase (*x*-axis), stimulation (active/sham) and montage (rAlC/lArC) conditions.

**FIGURE 7:**
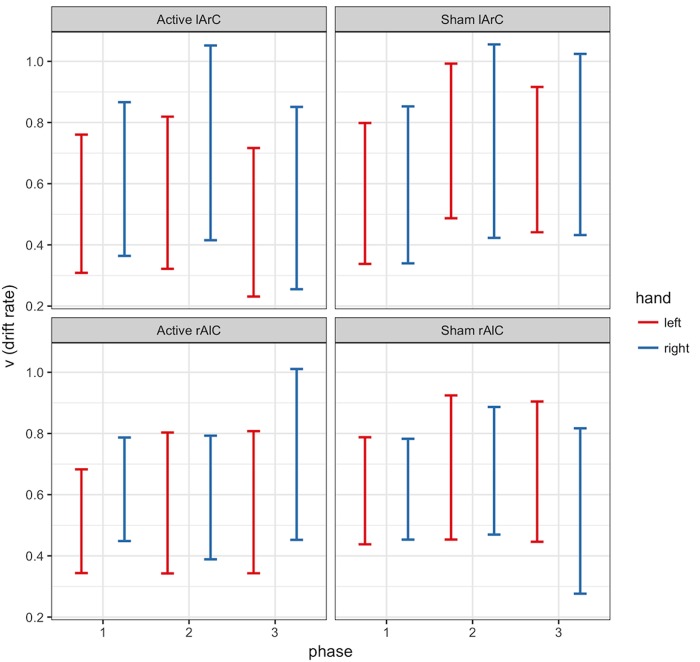
95% credible intervals of drift rate (𝒱) posterior distributions of left (red) and right (blue) hands for different phase (*x*-axis), stimulation (active/sham) and montage (rAlC/lArC) conditions.

**FIGURE 8:**
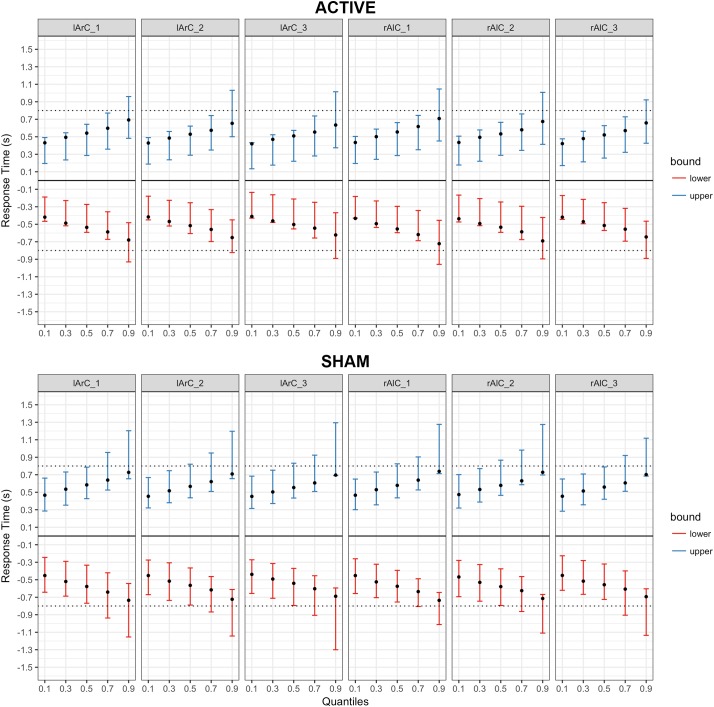
Posterior predictive checks for Model III (drift rate and non-decision time varying with the conditions) for responses with the right hand, comparing the estimated mean RTs per quartile to observed data. The bars represent the 95% credible intervals of the response time estimates for correct (upper bound; blue) and error (lower bound; red) responses, and the black dots stand for the observed mean RTs. Negative RTs are used to express the error responses. The dotted black line is the response deadline we imposed (0.8 s).

**FIGURE 9:**
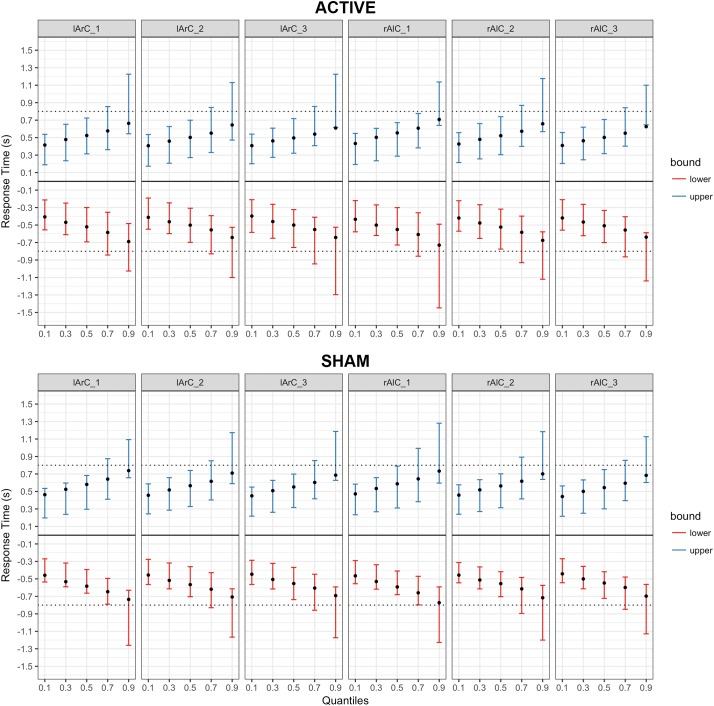
Posterior predictive checks for Model III (drift rate and non-decision time varying with the conditions) for responses with the left hand, comparing the estimated mean RTs per quartile to observed data. The bars represent the 95% credible intervals of the response time estimates for correct (upper bound; blue) and error (lower bound; red) responses, and the black dots stand for the observed mean RTs. Negative RTs are used to express the error responses. The dotted black line is the response deadline we imposed (0.8 s).

The model that allowed both non-decision time and drift rate to vary with condition, montage, and phase (M3) performed better than the null model (M0) and the model that let only non-decision time (M1) or only drift rate (M2) vary (for the left hand, Δ*DIC_M3-M0_* = -8741.32, Δ*DIC_M3-M1_* = -19.47, Δ*DIC_M3-M2_* = -8810.74; for the right hand, Δ*DIC_M3-M0_* = -10331.75, Δ*DIC_M3-M1_* = -26.82, Δ*DIC_M3-M2_* = -10415.99). In this model, none of the posteriors were significantly separated from each other for either parameter of interest and either hand (see **Figure 6** for *t_0_* and **Figure 7** for *v*; *P* < 0.95 for all comparison between stimulation conditions, electrode montage, and phases). The data generated from the parameter estimates of the model captured most aspects of the observed data with a few exceptions (**Figures 8, 9**). The most systematic discrepancy was observed in the mean standard deviation of both correct and error RTs in all conditions: the RT distributions in our data had systematically lower standard deviations than the data generated with the fitted model. However, the 95% credible intervals of the model-generated RTs managed to capture the observed RTs for all quantiles despite this difference in variance, demonstrating sufficient fit quality.

#### Signal Detection Theory Outcomes

**FIGURE 10:**
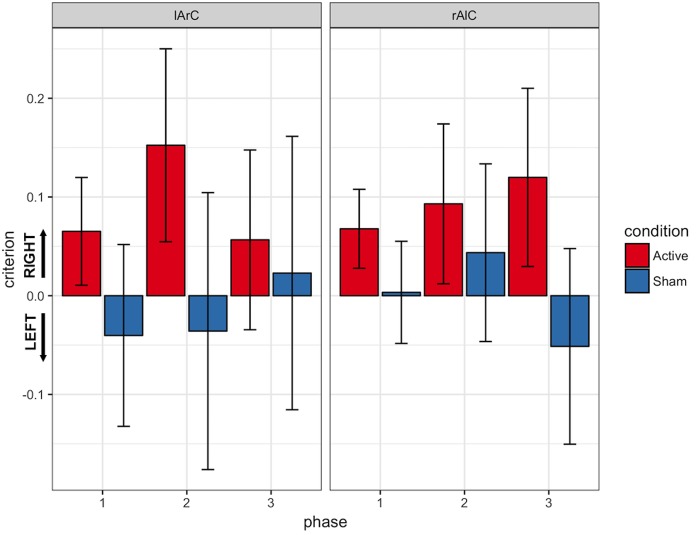
Response criterions of participants for different phase, stimulation (active/sham), and montage (lArC/rAlC) conditions. Responses with the right hand were considered to indicate “signal” and responses with the left hand were considered to indicate “noise”. Accordingly, a criterion above 0 indicates a bias toward the right hand and a value below 0 indicates a bias toward the left hand. Error bars represent standard error of mean.

**FIGURE 11:**
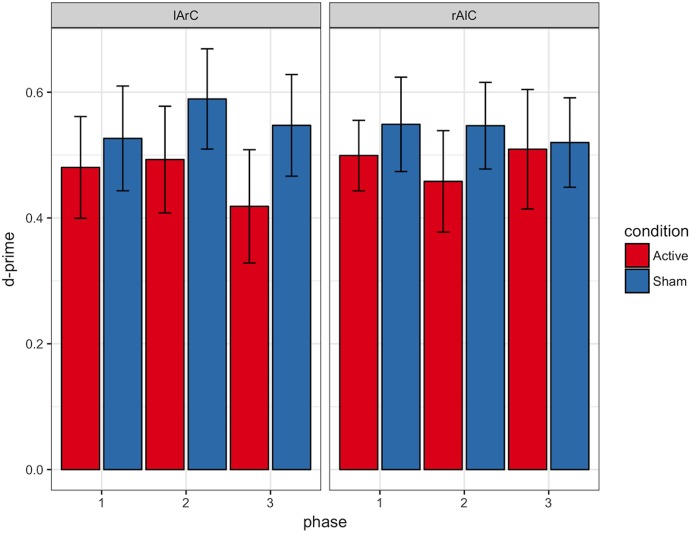
D-prime values of participants for different phase, stimulation (active/sham) and montage (rAlC/lArC) conditions. Error bars represent standard error of mean.

We provide the descriptive statistics of the Signal Detection Theory outcomes in **Figures 10, 11**. The three-way interaction between condition, montage, and phase was not significant either for the criterion [*F*(2,44) = 2.24, *p* = 0.12, *η^*2*^* = 0.09; *BF_exclusion_* = 7348] or d-prime [*F*(2,44) = 0.87, *p* = 0.42, *η^*2*^*= 0.04; *BF_exclusion_* = 3312].

## Discussion

In the present study, we applied tDCS over the primary motor cortices in order to assess the effects of motor cortex excitability at the level of both behavioral outcomes (choice behavior and response time) and latent variables of decision making [starting point bias (SDT’s criterion as a proxy), non-decision delay and drift rate]. We designed the study to include a conceptual replication of the first experiment in Javadi et al. (2015), using a different perceptual decision making task, adding a between-subjects sham control, and an additional phase to test the offline after-effects of tDCS. Despite the high effect size reported in the original study, we observed strong evidence indicating no effect of tDCS over the primary motor cortex on behavioral outcomes (hand choice or response time) in 2AFC.

Despite not modeling their data, Javadi et al. (2015) argued that their results are mostly in line with modified starting points in accumulation to bound theoretic approaches to decision making. We analyzed the data under different theoretical approaches (i.e., Drift Diffusion Model, Signal Detection Theory) that would approximately address Javadi et al. (2015)’s interpretation regarding the latent processes. Yet, our results did not confirm the induction of any bias or choice readiness due to tDCS manipulations. This failed replication adds to the evidence demonstrating inconsistent effects of tDCS on motor behavior (e.g., Antal et al., 2004; Boggio et al., 2006; Galea and Celnik, 2009; Tanaka et al., 2009; Horvath et al., 2014, 2016).

A number of reasons might have led to the observed discrepancy between the results of Javadi et al. (2015) and the current study. Despite the large degree of overlap in the task representation between the two studies (2AFC, static visual stimulus), our results might indicate that the effects gathered in Javadi et al. (2015) were peculiar to the stimulus used in that study (i.e., rectangles with different aspect ratios, presented for 100 ms). In other words, the effects gathered might be limited to the specific task used in Javadi et al. (2015) and thus might not be detectable by the grid-task. If this is the case, this would limit the generalizability of the inferences made based on Javadi et al.’s findings to decision making in general. In fact, the primary reason behind using a different task in the current study was to address the generalizability of the earlier findings to 2AFC.

Another change we introduced to the task in Javadi et al. (2015) was the response deadline, which is known to affect decision processes; mainly thresholds (Bogacz et al., 2010), but also evidence accumulation rates (Heitz, 2014) and motor processes (Spieser et al., 2017). We added the response deadline to counteract perceptual dominance by limiting the amount of evidence that could be gathered and increasing the relative contribution of motor excitability to the decision process. Javadi et al. (2015) achieved this using a very brief fixed viewing time (100 ms), but we chose to have the stimulus present throughout the trial in order to keep evidence accumulation constant during the trial (to better meet the DDM’s corresponding assumption). The participants had reasonable accuracy, indicating that they accumulated enough evidence within the limited time. Thus, the strength of the perceptual signal might have not left enough window for the effects of tDCS on motor behavior, especially hand choice. Since Javadi et al. (2015) did not report the overall accuracy level of participants, we cannot ascertain whether such an interpretation explains the discrepancy of our results and theirs.

Task difficulty is another factor that is different between the two studies, but we argue that this cannot fully account for the discrepancy between our results and the original study. Not all conditions in Javadi et al.’s (2015) task were comparable to ours in terms of difficulty (i.e., in the original study, the aspect ratio was very high in certain conditions making the task simple in those trials). We were concerned about the impact of both too-easy and too-difficult trials. Trials that were too difficult, especially under a response deadline (or brief fixed viewing time, as in Javadi et al., 2015), would confuse and frustrate participants and thus contaminate the data with noise that we could not account for. Trials that were too easy, on the other hand, would make the slight motor stimulation/inhibition irrelevant for the obvious response and render expectations of an effect on hand choice unrealistic. Participants had considerably high levels of accuracy given the difficulty of our task (**M*_accuracy_* = 63.7%, *range*: 50.5–76.3%), and performed above chance level at baseline (*p* < 0.05 for binomial test for each participant in both sessions, with three exceptions where the participants were above chance level at the baseline of one session but not the other). Furthermore, there are instances in literature where tDCS could not manipulate motor behavior even in very simple paradigms such as simple reaction time tasks (e.g., Horvath et al., 2015, 2016), or required a large sample size (*n* = 75) to show such an effect due to low effect size (Minarik et al., 2016), underscoring the idea that difficulty or complexity of the task alone cannot fully account for the failure of detecting an otherwise detectable effect.

Another deviation from the original study was the use of a larger stimulation surface area in our study, which would result in a lower stimulation density. Stimulation density is one of the major factors that has been shown to change the effect size in tDCS studies (Mancuso et al., 2016). However, Kidgell et al. (2013) shows no effect of intensity (and density, as they use same size electrodes with varying current intensities) on motor excitability, and many studies show significant motor effects of tDCS with a lower density than the current study (e.g., Antal et al., 2004; Boggio et al., 2006; Kuo et al., 2008; Stagg et al., 2011; Tanaka et al., 2009). This suggests that our null results cannot be explained solely by the size of the surface area. We do not think sample size was a factor behind not being able to replicate the earlier findings either given the results of our *a priori* power analysis (see section “Materials and Methods”). Briefly, the procedural differences between Javadi et al. (2015) and the current study are not likely the sources of the discrepant findings.

Another extension of the current study was the use of sham-control. The absence of a sham-control raises issues not only in terms of blinding but also due to the inter-individual variability in physiological responses to tDCS, which decreases the validity of the studies and complicates the interpretation of inconsistent findings in literature (see Li et al., 2015). Our double-blind sham-controlled design aimed to overcome this caveat but the between-subject nature of stimulation condition factor (active vs. sham) renders our study still susceptible to the effects of inter-individual variability in participants’ responses to tDCS (Fricke et al., 2011; for a review, see Horvath et al., 2014; Nitsche et al., 2009). Yet, if inter-individual variability is the sole factor that has nullified the established effect, this reinforces the already existing concern regarding the applicability and efficacy of tDCS.

As a further extension to Javadi et al. (2015), we added an additional phase to test the short-term offline after-effects of tDCS. There is evidence that motor-cortex tDCS is capable of inducing relatively long-lasting changes; stimulation in the range of seconds only induces excitability changes during the stimulation while stimulation lasting from 9 to 13 min may create a change that lasts approximately for an hour (Nitsche and Paulus, 2000, 2001; Nitsche et al., 2003). This, along with the findings that NMDA antagonists prevent the long-lasting effects of tDCS (Liebetanz et al., 2002; Nitsche et al., 2003), suggests that the effects induced by tDCS and long-term potentiation may be sharing similar mechanisms. Following these findings, we hypothesized that the effect we aimed to replicate would extend into the third phase as well. Yet, consistent with the lack of a difference during the stimulation phase (second phase), we could not establish an offline after-effect in the third phase either.

Another potential reason for the discrepancy of our findings with Javadi et al. (2015) could be the differences in sample characteristics since we introduced a criterion for selecting participants. Eligibility criterion required participants to not display any sort of bias toward one of the stimulus features (e.g., due to the participant preferentially attending to one color over the other) or a bias for executing the response with one of the hands regardless of stimulus characteristics. Following these criteria, we excluded 40% of the recruited participants in a preliminary session. This, we believe, increases the rigor of our experimental design and thus, reliability of our results. First, any motor bias introduced via primary motor cortex stimulation could interact in unexpected ways with perceptual or attentional biases. Second, the susceptibility of hand-choice to primary motor cortex stimulation would differ for people who already have a bias for a particular hand. Since our main aim was to induce a hand-choice bias, we only recruited participants who did not have inherent biases, which created a relatively uniform sample. However, we do not think that the effects in Javadi et al. (2015) arose solely due to such biases since the bias could go both ways (white/black or right/left) and the response-to-hand mappings were counterbalanced across participants.

We extensively exercised fitting the behavioral data both with the drift-diffusion model (DDM) and linear ballistic accumulator (LBA) model in order to estimate the psychologically meaningful latent variables that were of theoretical interest to us (i.e., starting point, non-decision related delay, and drift rate). Although the LBA model provided good fits to the data, the estimated non-decision related delays were too short to be considered psychologically plausible (e.g., *t_0_* = 87.84 ms, range = 0–576 ms), particularly given the variable response-to-stimulus intervals. The hierarchical DDM provided sufficient fits for the models with non-decision time and drift rate estimates, but not for the starting point estimates (details of the problems with these models are discussed in the “Materials and Methods” section). These have reduced the credibility of the use of the problematic models in the context of the current data. These being said, none of the comparisons of the non-decision related delays, drift rates, and starting points estimated from model fits revealed significant differences that were in line with the previous findings of Javadi et al. (2015). Future studies can employ tasks without speed instructions (free-response as opposed to deadline) that may yield RT distributions with more skew to better meet the assumptions of the model and better account for the decision outputs within the framework of decision theoretic approaches.

Another arguable caveat of this study would be the possibility that our sham stimulation had a true physiological effect, which would reduce the difference between active and sham conditions in terms of their effects on hand choice or RTs. However, our null findings cannot be solely explained by this factor. In this experiment, we investigated how RT and hand choice changes from the first phase to the second and third as a function of electrode montage (rAlC/lArC; within-subject) and stimulation conditions (active/sham; between-subject). Active/sham comparisons were relevant for our task, but it was not for Javadi et al. (2015), since they only traced how different electrode montages under active stimulation influences the behavior in comparison to a baseline phase, without any active-sham comparisons. Thus, even if we would assume that sham stimulation has a true physiological effect, we would at least expect to replicate the effects that are reported by Javadi et al. (2015), which did not rely on active-sham comparisons. Active stimulation lasted 30 times longer than the sham stimulation. The only purpose of the sham-stimulation was to give the participant a sensation of normal-like stimulation, which would reduce any possible placebo effects. If sham stimulation indeed had true physiological effects, considering the vast difference in stimulation longevity, we would still expect any effect to be greater under active stimulation. We would also like to point out instances in literature that demonstrated equivalent sham protocols producing comparable results with no-stimulation conditions (e.g., Antal et al., 2008; Marangolo et al., 2013).

The absence of an effect of tDCS over the primary motor cortex on motor behavior presents an interesting parallelism to the differential findings regarding the effect of tDCS vs. transcranial magnetic stimulation (specifically, theta burst stimulation) of the right preSMA on decision threshold settings. Two recent studies have shown that the inhibition of right preSMA via continuous theta burst stimulation (Tosun et al., 2017; Berkay et al., 2018) leads to higher decision threshold settings whereas the stimulation of the same brain area via intermittent theta burst stimulation (Berkay et al., 2018) leads to lower decision threshold settings. Interestingly, however, in three independent studies Hollander et al. (2016) showed that tDCS of pre-SMA by the placement of the anodal electrode on FCZ did not produce any effects on threshold settings. Consequently, tDCS might simply not be providing strong enough modulation of the cortical excitability to exert detectable effects on the decision processes (at least not in healthy participants). Future studies that use similar paradigms with transcranial magnetic stimulation of the primary motor cortices would shed light on the differential efficacy of these methods in modulating behavior.

## Conclusion

To conclude, this study investigated the effects of primary motor cortex tDCS on response latencies and choice bias with the contralateral hand. We modeled the experimental paradigm as a conceptual replication of Javadi et al. (2015) with minor extensions but could not replicate their findings of modified hand-choice and response latencies with the hand associated with the stimulated region. Task type, difficulty, or the size of the stimulation area might be among the factors that have contributed to the null findings. If such task-related differences indeed were responsible for the null results, this raises concerns regarding the experimental applications of tDCS and the generalizability of the results to real-life settings at least in individuals with normative cognitive functioning.

## Data Availability

The datasets analyzed for this study can be found in the Open Science Framework repository [osf.io/uys2k].

## Author Contributions

ET, SA, BA, CG, and FB contributed to study design. BA coded the computerized task. ET, SA, BA, and CG collected the data. ET organized the data. ET, SA, and BA contributed to data analysis. FB and YÇ provided guidance in interpreting the results. All authors wrote and edited parts of the manuscript and approved the submitted version.

## Conflict of Interest Statement

The authors declare that the research was conducted in the absence of any commercial or financial relationships that could be construed as a potential conflict of interest.
